# Steroid-Responsive Encephalopathy Associated with Autoimmune Thyroiditis Presenting as Confusion, Dysphasia, and Myoclonus

**DOI:** 10.1155/2012/782127

**Published:** 2012-06-13

**Authors:** S. A. Ryan, C. Kennedy, H. J. Harrington

**Affiliations:** Department of Neurology, Mercy University Hospital, Cork, Ireland

## Abstract

Steroid response encephalopathy associated with autoimmune thyroiditis (SREAT), or Hashimoto's encephalopathy, is a rare disorder believed to be immune-mediated. It is most often characterized by a subacute onset of confusion with altered level of consciousness, seizures, and myoclonus. We describe the case of a 48-year-old gentleman who presented with confusion and dysphasia. Specific clinical features and laboratory results led to a diagnosis of Hashimoto's encephalopathy. This case highlights the core features of this condition and the potential for complete response to steroid therapy.

## 1. Introduction

Steroid response encephalopathy associated with autoimmune thyroiditis (SREAT) is a rare disorder first described in 1966. It can present acutely with multiple recurrent focal neurological events or with a progressive, diffuse pattern characterized by cognitive impairment. It is under-recognized by physicians, and the purpose of this report is to add to the literature and highlight the potential for excellent clinical outcome with appropriate therapy.

## 2. Case Presentation

A 48-year-old gentleman presented to the emergency department with a six-hour history of confusion and dysphasia. He was a smoker of five cigars a day and consumed 40 units of alcohol per week. His past medical history was notable for an episode of pancreatitis. His son had type 1 diabetes mellitus; family history was, otherwise, unremarkable.

His wife described two discrete episodes of bizarre behaviour in the year prior to his current admission. She reported behaviour such as shaking salt into his tea and mixing up various household utensils. The first of these episodes lasted three days and was associated with ataxia. The second, six months later, was associated with an expressive dysphasia. Investigations at that time included routine bloods, ANA, ANCA, CT brain, MRI brain, and EEG, all of which were normal. A lumbar puncture during the second episode yielded CSF with normal cytology but an elevated protein of 1 g. His work colleagues also reported some intermittent unusual behaviour and subtle personality changes in recent months. His clinical condition improved spontaneously on both of these occasions and a presumptive diagnosis of transient ischaemic attacks was made.

When reviewed in the emergency department on this occasion, he was agitated. Vital signs were normal. He had an expressive dysphasia and appeared disorientated and confused scoring 26/30 on the minimental state score (MMSE). Cranial and peripheral nerve examinations were normal, as was fundoscopy. There was no meningism. Cardiorespiratory and abdominal examinations were normal. Initial investigations revealed normal full blood count, inflammatory markers, electrolytes, and renal function. Liver function tests, alpha-feto protein, and ammonia levels were normal. CK was mildly elevated at 211. CT brain and MRI brain using a 1.5 Tesla machine were normal. CSF analysis revealed an elevated protein of 0.81 with normal cytology and glucose. EEG revealed intermixed theta activity. Over the following days, he developed myoclonus, a marked startle reflex, diffuse rigidity, hyperreflexia, and a fine tremor. He also developed generalised seizures.

Further laboratory tests including VDRL, TPHA, ANCA, anti-GBM, heavy metal screen, antineuronal antibodies, red cell transketolase, brucella, mycoplasma, and lyme serology were all normal. Autoimmune screen and haematinics including vitamin B12 levels were likewise normal.

Thyroid function showed T4 10.3 and TSH 9.97 with markedly elevated anti-thyroid peroxidase antibodies at 25600 and normal antithyroglobulin antibodies. A diagnosis of Hashimoto's encephalopathy was made and high-dose steroids initiated. Within days, there was marked clinical improvement with full resolution of confusion and myoclonus. He was discharged well several days later on high-dose oral prednisolone. This was gradually reduced over six months, without relapse. One year after this hospital admission, routine thyroid function tests revealed overt hypothyroidism with T4 of 7.4 and TSH of 51. At this point, thyroxine replacement was introduced.

## 3. Discussion

Hashimoto's encephalopathy remains a controversial entity since its first description in 1966 [1]. The exact aetiology has yet to be elucidated although is assumed to be autoimmune. Current opinion is divided on the exact nature of this autoimmune response. Primary demyelination, vasculitis, immune complex deposition, and direct antibody-mediated neuronal injury have all been proposed as mechanisms of disease. It is characterised by confusion with or without myoclonus, seizures, hyperreflexia, and psychosis. Presentation may be an insidious development of cognitive impairment or recurrent acute episodes of focal neurological deficit with confusion [2].

Laboratory findings are largely nonspecific. There is typically a raised CSF protein level with normal glucose with or without a lymphocytic pleocytosis. Nonspecific EEG abnormalities usually comprising slowing of background activity are seen in the majority of patients, whilst focal spikes or sharp waves are less common [3]. There are reports of EEG abnormalities recovering rapidly with steroid therapy while other cases illustrate a lag between EEG improvement and clinical improvement [4]. Magnetic resonance imaging (MRI) may be normal or demonstrate nonspecific T2 signal abnormalities in the subcortical white matter that do not enhance with gadolinium. In rare cases, diffuse white matter changes or meningeal enhancement have been described [2]. Imaging using higher field magnets such as 3T MR scanners may detect subtle abnormalities not seen on the 1.5T machine used in our case. Thyroid dysfunction may be present. The most striking abnormality is raised antithyroid antibodies. Antithyroid peroxidase antibodies are markedly elevated with or without elevated antithyroglobulin antibodies [4].

Initial treatment is with high-dose steroids titrated according to clinical response [5]. Thyroid dysfunction should also be appropriately treated if present. Response to steroid therapy is usually excellent and is followed by slow steroid tapering and eventual discontinuation. Steroid intolerant patients may respond to cyclosporine or azathioprine. There are also several case reports describing good clinical outcomes following plasma exchange [6] and intravenous immunoglobulin [7]. It is unclear whether this condition is directly related to thyroid disease or whether the relationship is due to differential expression of an autoimmune phenotype. The term steroid-responsive encephalopathy associated with autoimmune thyroiditis (SREAT) is, therefore, preferred by some authorities. The long-term neurological prognosis in Hashimoto's encephalopathy is good. Persistent cognitive deficits develop in a minority and are more likely if treatment is considerably delayed [8].

There remains much to be discovered regarding the pathogenesis of Hashimoto's encephalopathy. It should be considered in the differential diagnosis of any metabolic encephalopathy as prompt treatment can lead to excellent recovery.
